# Rapid Design and Delivery of an Experience-Based Co-designed Mobile App to Support the Mental Health Needs of Health Care Workers Affected by the COVID-19 Pandemic: Impact Evaluation Protocol

**DOI:** 10.2196/26168

**Published:** 2021-03-09

**Authors:** Matthew Lewis, Victoria J Palmer, Aneta Kotevski, Konstancja Densley, Meaghan L O'Donnell, Caroline Johnson, Franz Wohlgezogen, Kathleen Gray, Kate Robins-Browne, Luke Burchill

**Affiliations:** 1 Integrated Mental Health Research Program Department of General Practice, Melbourne Medical School The University of Melbourne Melbourne Australia; 2 Centre for Digital Transformation of Health The University of Melbourne Melbourne Australia; 3 Department of Medicine The University of Melbourne Melbourne Australia; 4 Phoenix Australia Department of Psychiatry The University of Melbourne Melbourne Australia; 5 Centre for Workplace Leadership Faculty of Business and Economics The University of Melbourne Melbourne Australia; 6 Department of Medicine The University of Melbourne Parkville Australia; 7 Department of Cardiology Royal Melbourne Hospital Parkville Australia

**Keywords:** mental health, mobile applications, COVID-19, health personnel, experience-based co-design, impact, evaluation, digital interventions, app, intervention, health care worker, design, delivery, support

## Abstract

**Background:**

The COVID-19 pandemic has highlighted the importance of health care workers’ mental health and well-being for the successful function of the health care system. Few targeted digital tools exist to support the mental health of hospital-based health care workers, and none of them appear to have been led and co-designed by health care workers.

**Objective:**

RMHive is being led and developed by health care workers using experience-based co-design (EBCD) processes as a mobile app to support the mental health challenges posed by the COVID-19 pandemic to health care workers. We present a protocol for the impact evaluation for the rapid design and delivery of the RMHive mobile app.

**Methods:**

The impact evaluation will adopt a mixed methods design. Qualitative data from photo interviews undertaken with up to 30 health care workers and semistructured interviews conducted with up to 30 governance stakeholders will be integrated with qualitative and quantitative user analytics data and user-generated demographic and mental health data entered into the app. Analyses will address three evaluation questions related to engagement with the mobile app, implementation and integration of the app, and the impact of the app on individual mental health outcomes. The design and development will be described using the Mobile Health Evidence Reporting and Assessment guidelines. Implementation of the app will be evaluated using normalization process theory to analyze qualitative data from interviews combined with text and video analysis from the semistructured interviews. Mental health impacts will be assessed using the total score of the 4-item Patient Health Questionnaire (PHQ4) and subscale scores for the 2-item Patient Health Questionnaire for depression and the 2-item Generalized Anxiety Scale for anxiety. The PHQ4 will be completed at baseline and at 14 and 28 days.

**Results:**

The anticipated average use period of the app is 30 days. The rapid design will occur over four months using EBCD to collect qualitative data and develop app content. The impact evaluation will monitor outcome data for up to 12 weeks following hospital-wide release of the minimal viable product release. The study received funding and ethics approvals in June 2020. Outcome data is expected to be available in March 2021, and the impact evaluation is expected to be published mid-2021.

**Conclusions:**

The impact evaluation will examine the rapid design, development, and implementation of the RMHive app and its impact on mental health outcomes for health care workers. Findings from the impact evaluation will provide guidance for the integration of EBCD in rapid design and implementation processes. The evaluation will also inform future development and rollout of the app to support the mental health needs of hospital-based health care workers more widely.

**International Registered Report Identifier (IRRID):**

DERR1-10.2196/26168

## Introduction

### Background

The mental health and well-being of health care workers should be a major public health priority [1] both during the COVID-19 pandemic and beyond to support the successful functioning of the health care system. Health care workers, particularly nurses and physicians, experience significant mental health challenges; these have been exacerbated during the early stages of the COVID-19 pandemic, with high rates of depression (23.2%), anxiety (22.8%), and insomnia (38.9%) reported [2]. Similarly high rates of clinically significant anxiety (45%), depression (38%), and posttraumatic stress disorder (19%) have been observed during and following significant viral outbreaks and pandemics [3]. Despite the recognized impact of pandemics on the mental health and well-being of health care workers, few digital solutions have been developed and delivered to support the mental health and well-being of hospital-based health care workers. A mobile app could provide readily available, evidence-based support for stress management, mental health, and well-being to hospital-based health care workers during the COVID-19 pandemic. By using experience-based co-design (EBCD) approaches, this app could deliver appropriate supports across professional groups.

Health care workers are often reluctant to seek appropriate and timely mental health support [4]. Perceived stigma and career impact, time challenges, and a work culture that values stoicism are recognized as contributing barriers to help-seeking [4-6]. Without support, all health care workers are at increased risk of major mental health complications [4,7]. Targeted approaches to maintain and improve the mental health of health care workers are needed that can address barriers to help-seeking and meet health care workers “where they are” according to their professional needs. A mobile app co-designed by health care workers offering mental health support may have potential as a readily accessible, cost-effective, evidence-based, and scalable tool to achieve these goals [8].

The more a mobile app integrates the lived experiences of health care workers through the use of co-design processes, the more likely it will be to respond to their needs and ideally lead to increased uptake and engagement. While digital health interventions and mobile app development use human-centered design (HCD) principles to meet user needs [9], few embed and follow an EBCD approach. EBCD offers power sharing and approaches that can further extend HCD methods to develop deep insights that enable users to actively shape and co-design solutions [10]. EBCD has typically been used in health care quality improvement as a method for staff, patients, and carers to collaborate on the design of solutions drawing on narrative and story-based approaches through interviews, film, or other visual methods, such as digital stories, coupled with facilitated co-design using design thinking and participatory approaches. The integration of the lived experience of end users via EBCD processes will ensure that outcomes are targeted and co-designed with those most likely to be impacted by an issue, change to process, or intervention [11,12]. EBCD provides an avenue to more deeply embed end users’ experiences as the primary driver of change processes and provides a commitment to shared power arrangements and decision-making not currently addressed by the adoption of HCD principles alone [10]. The explicit use of EBCD as a way to embed lived experience will ideally result in greater uptake of the mobile app and increased engagement. Several digital mental health support responses for health care workers have been developed to address the impact of COVID-19 [13], and while some were led, peer-designed, and delivered by health care workers, we were unable to identify any that were co-designed by and for health care workers themselves. Addressing this gap may be part of what is required to improve overall uptake, engagement, and use of mobile apps by individuals. The RMHive app will attempt to do this by embedding health care workers in the design, development, and implementation of a mobile app using EBCD.

Integrating the lived experience of health care workers in the design and development of mobile apps is important to support their mental health needs [12]. Equally important is a systematic evaluation of the development process and outcomes that can be used to support replication of mobile apps or digital interventions. This includes outlining the underlying theoretical frameworks, clear overviews of the design, development, and implementation, and documentation of impact [8,14]. Existing evaluations focus on the development and implementation of mobile apps largely from a technological perspective; however, they can take a more limited evaluation of the wider contextual, organizational, and individual factors that influence uptake and impact on outcomes [15]. A broader impact evaluation approach enables sociotechnical digital health perspectives to be considered in the context of intersecting factors and causal attributions across the design, development, and implementation continuum for programmatic scale-up [16].

This protocol describes the impact evaluation of the RMHive mobile app, which is being led by health care workers as an EBCD intervention for health care workers with mental health needs arising from the COVID-19 pandemic. Using EBCD, health care workers from different professional groupings will serve as co-design partners across all phases of research, from ideation to implementation. RMHive is not being developed as a clinical or therapeutic tool. The impact evaluation will integrate data from the design and development and the wider implementation of RMHive and lead to the formulation of a theory of change that can explain both positive and negative impacts to guide future delivery of the mobile app across other health services and hospital networks.

### Objectives

A rapid design and development cycle will be adopted using EBCD to identify experiences and create an app prototype within 3-4 months to support the mental health needs of hospital-based health care workers working in the COVID-19 context. The adopted approach is analogous to rapid prototyping in design thinking [17] through the use of rapid co-design processes. The impact evaluation addresses three questions using quantitative and qualitative data that will be collected across all stages:

1. What is health care workers’ engagement with RMHive, including use patterns, perceptions of content, and overall level of engagement?
2. What contextual, socio-technical, organizational, and individual features support or hinder implementation of the RMHive app?
3. What are the identifiable impacts on the mental health of individual health care workers through adoption, implementation, and use of the RMHive app?

This protocol will describe the planned design, development, and implementation of the RMHive mobile app using EBCD. It will describe the data to be collected to inform the impact evaluation and the proposed analysis plan.

## Methods

### Setting and Locations

RMHive will undergo development, beta testing, and implementation with hospital-based health care workers across Royal Melbourne Hospital (RMH). RMH is a large tertiary referral hospital that includes two major campuses, an acute care city campus and a second inner city campus providing subacute services, including aged care; both campuses have been heavily impacted by the COVID-19 pandemic. RMH is the largest provider of mental health services in Victoria, with services spanning the northern and western suburbs of Melbourne. RMH is a part of the Melbourne Health Network, which reported 10,000 staff and volunteers in 2018/2019. In 2019, there were 79,799 presentations to the emergency department and 105,493 inpatient admissions [18]. Design, development, and beta testing will be conducted with the emergency and cardiology departments as two settings impacted by the COVID-19 pandemic. Following completion of the beta test phases and refinements arising from this process, RMHive will be implemented across RMH and made available to all health care workers hospital-wide. Although it is recognized that some context- and profession-specific needs may remain unaddressed in this preliminary rapid design process, a decision was made to perform a hospital-wide rollout of the app to explore use patterns and gather feedback to inform future iterations and refinements of the app. Ethics approval for this study has been provided by the University of Melbourne Human Research Ethics Committee (Ethics ID #2056866).

### Overview of the RMHive Design, Development, and Implementation Process

**Figure 1:**
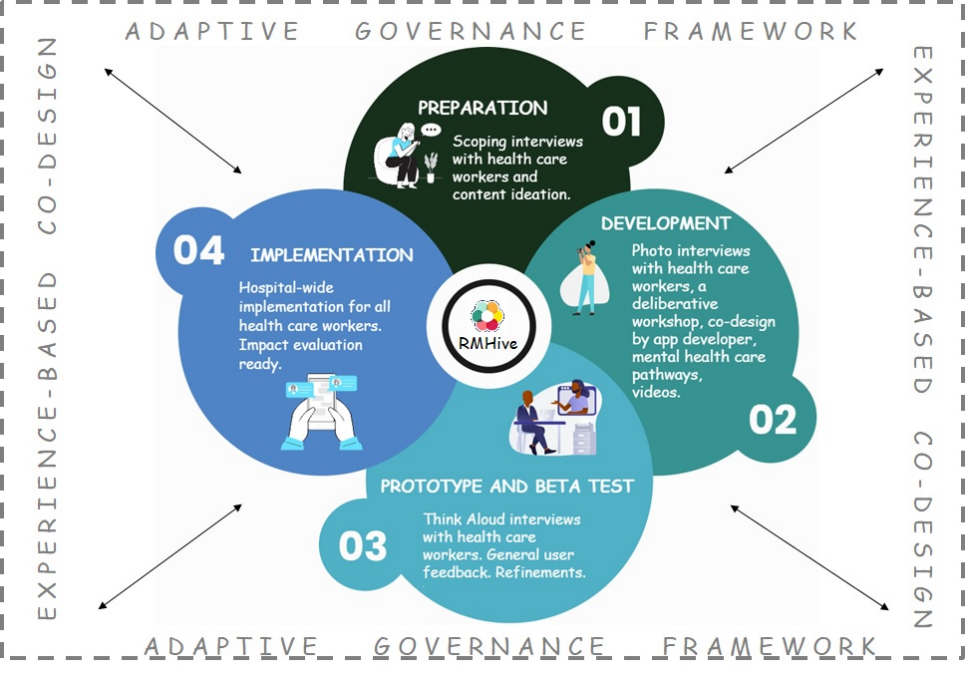
Planned design, development, and implementation framework of the RMHive app.

The project will adopt a rapid design, development, and implementation process using EBCD to progress from needs identification of health care workers and content creation through to app design and development and beta testing to a minimally viable product (MVP) release. An overview of the design, development, and implementation framework is shown in Figure 1.

#### Project and Governance Team

An important governance principle for the research team will be adaptiveness to ensure that the design and implementation process is sufficiently flexible to adjust to complex, unpredictable changes that may occur while undertaking the research (eg, due to emerging events throughout the COVID-19 pandemic). An adaptive governance framework will be adopted to support the design, development, and implementation of RMHive [19]. Adaptive governance provides a structured approach for decision-making in complex and dynamic systems while paying attention to the needs of grassroots community members [20]. Key to adaptive governance is shared decision-making arrangements that complement the collaborative and power-sharing goals of the EBCD approach. When applied to RMHive, adaptive governance is being used to ensure that health care workers from diverse professional backgrounds, including clinical workers (eg, nurses, physicians, psychologists, primary care specialists) and nonclinical workers (eg, administrative staff, environmental service assistants) are continually involved in shared decision-making and feedback across all phases of research and according to the changing community needs of health care workers. This extends to providing mechanisms of accountability of the research team in the EBCD processes.

The co-design and implementation of the RMHive app will be managed by a health care worker–led project team and will include health care workers in multiple project governance roles and as advisory committee members. Health care worker members of the project team and advisors will be invited to participate through posters and flyers posted in staff common rooms and drawn from critical care emergency and cardiology units for the first rapid design phases. It is anticipated that the health care workers will most commonly be nurses and physicians, as these are the two largest professional groups in the hospital. The health care worker advisory group will provide input and feedback and share decision-making throughout the project. The adaptive governance framework supports feedback loops that ensure health care worker input is reviewed on a continuing basis, with decision-making shared by health care workers on the project team meeting every 2 weeks. The project team will be multidisciplinary and incorporate experts in EBCD, implementation science, evaluation, applied ethics, mental health clinical care, project governance, mental health care research and delivery, mobile app development, digital health interventions and evaluation, and creative content production.

#### Preparatory Phase

Prior to commencement of the co-design work, scoping interviews were conducted during the initial wave of COVID-19 in early 2020 with a small but diverse group of health care workers (N=6). These interviews were conducted to understand the experiences and needs of health care workers working during the pandemic. Semistructured interviews included scoping questions codeveloped by health care workers and the researchers leading the study (available upon request). The health care workers reported being overwhelmed with written communication (guidelines, clinical directives, news reports) and expressed a clear preference for video- rather than text-based information. Using these experiences, the project team generated ideas for video-based content that reflected health care workers’ experiences but that also responded to their needs. These were (1) mental preparation and coping strategies; (2) remaining connected through hearing other experiences of working on the front line; and (3) having a “time out”/a break from the “dark bubble” they were living in. The broad content themes were pitched to health care worker members of the implementation team and led to a decision to develop video content within the app.

#### Needs Analysis and Understanding Context

Leveraging the expertise of EBCD researchers working on the project team, health care workers selected photo interviews (photo elicitation) as a relevant method for understanding the mental health needs and lived experiences of health care workers. We have previously shown photo elicitation to be a relevant participatory visual method that can be successfully applied across different contexts and research settings [21]. Photo interview methods enable a narrative approach to identifying needs and understanding context [21] and can help people to articulate complex concepts, discuss sensitive topics, and provide insight into the inner worlds of feelings, emotions, and thoughts [22]. Up to 30 health care workers from the emergency and cardiology departments will be invited to participate in the photo interviews [21]. The choice of these departments was based on the professional groups representing the largest proportions of hospital staff.

The photo interview method will involve health care workers being asked to provide the research team with a series of 3-5 digital images produced on smartphones reflecting their experience of the COVID-19 pandemic, including challenges associated with working on the front line as well as experiences or behaviors that are helping them to meet these challenges. The images will inform a semistructured telephone interview to draw out key “touchpoints” related to the experiences of health care workers during the pandemic and the impacts on their mental health. A touchpoint is a term used in EBCD to refer to the places and the ways in which a person comes in touch with a particular service or organization, or how an issue touches them directly (eg, the subjective world of the person) [23]. The touchpoints will be further explored in a deliberative workshop to scope content areas to be developed within the app. The workshop discussion will be analyzed to identify one or two key touchpoints that will inform an additional co-design workshop to be conducted by the app developers, Curve Tomorrow (explained below).

Early in the RMHive development process, a series of interviews will be conducted with a diverse range of hospital managers and leaders within RMH (N=30) to gain insights into existing mental health programs, systems, organizational context, and services for the mental health of health care workers. It is likely that this group will be involved in initiatives for mental health, and this interview data will provide further contextual data to inform the implementation of RMHive and its impact evaluation.

#### Content Creation

Video content will be developed by a university-based video producer working with a team of externally based creatives, including filmmakers, animators, and script writers, based on the expressed needs and specific questions posed by health care workers in the preparatory phase, which resulted in the creative team receiving health care worker approval for four planned video series. The first series of videos will feature health experts answering questions posed by health care workers working on the front line during the COVID-19 pandemic. The second series will include animated videos developed in conjunction with Phoenix Australia, national experts in trauma-informed care, that focus on resilience and coping strategies (an issue that was raised by health care workers in the preparatory phase scoping interviews). Series 3 will include short videos based on themes identified by health care workers and that take a humorous tone based on feedback from health care workers that they needed a mental break from the daily challenges of the COVID-19 pandemic. The final series will be short films that use the audio taken from the photo interviews that address health care workers’ need to connect with others, and the filmmakers will creatively reflect on their experiences. Health care workers identified the experience of discussing their work lives and challenges as cathartic, and the content creation team has engaged eight filmmakers who will be asked to respond to each audio interview with a short film in an attempt to maintain a creative dialogue between the health care workers and creative team. Closing the communication loop, the health care workers will then respond to the short film with a written reflection. The films and responses are both planned to appear in the app.

A team of mental health clinicians will develop information about clinical support pathways and curate existing digital and face-to-face mental health support services and tools to be included in the RMHive app. These resources will match the touchpoints identified in the photo interviews with health care workers. The goal is to provide targeted pathways for mental health support depending on the needs of users in a simple, private, and focused way in response to need. Engagement and feedback of health care workers in the preparatory phase scoping interviews of the study identified that options to self-monitor mental health would be valuable. This self-monitoring is intended as a reflective point for individuals and to provide personalized engagement opportunities in the app for the user.

The mental health team determined that the Kessler Psychological Distress Scale (K10) [24], a measure of distress commonly used by general practitioners and in mental health settings, would be a useful tool to monitor mental health within the app. Self-monitoring mental health using mobile apps is an established effective method to increase emotional self-awareness [25]. Four general health and wellbeing questions will also be provided to prompt self-reflection regarding whether users are on track across four domains: mood, relationships, physical health, and productivity. In the absence of an evidence-based self-tracking tool, the four general health questions were codeveloped through consensus discussions with the project lead (LB) and mental health stream leads (MO, CJ) and the health care worker advisory group. The goal was to provide a simple, self-monitoring check-in option that could be completed as often as a user chose.

In addition to self-monitoring tools, the app will include the 4-item Patient Health Questionnaire (PHQ4) as a measure of symptom burden, functional impairment, and disability. The PHQ4 comprises the 2-item Patient Health Questionnaire (PHQ2) for depression and the Generalized Anxiety Scale-2 (GAD2) as brief, validated subscales [26]. These questionnaires will help to establish a baseline of the mental health needs of the health care workers (eg, impact evaluation question 1) and will be used to identify any changes to these needs during and post app use (eg, impact evaluation question 3).

#### RMHive Development, Beta Testing, and Refinement

A co-design workshop will be conducted by the app developer and industry partner, Curve Tomorrow, using HCD principles and building on the data collected from the preparatory scoping interviews, needs analysis, and content creation stages. This workshop will lead to a clickable prototype of RMHive that will be used to gather additional user feedback prior to the beta test version of the app. Curve Tomorrow will gather telephone feedback from health care workers about the clickable prototype to generate the beta test app version. The beta test version will be released to one hospital unit (the emergency department), and feedback from users will inform the refinements that are needed for the implementation of the MVP for release for health care worker use hospital-wide.

Think aloud interviews [27] will be conducted with a subsample of users during the beta testing to inform refinements and identify bugs and required fixes. Think aloud interviews are commonly used to test new products and technologies, as simulated situations, and to enable a user to express their thoughts and feelings out loud as they use the app in real time [28]. The think aloud interviews will be audio- and video-recorded, and the outcomes will be summarized for beta test changes and reported in the impact evaluation. Key themes related to usability and perception of the content will be explored. An opt-in process to participate in the think aloud interviews will be employed.

### App Architecture

RMHive will be designed to be a stand-alone app and will not be integrated into existing data repositories, human resource records, or electronic medical records. The RMHive app will be developed using the Ionic app development framework with a Rails backend and will function on iOS and Android operating systems. The app will be hosted via the secure cloud application platform Heroku. Data entered within the app will be anonymous to the research team, and only deidentified data will be provided to the research team for analysis. In the Terms of Use, app users will be asked to provide consent for the research team to contact them for think aloud interviews and collect data for the impact evaluation. It is planned that RMHive will be incorporated as a stand-alone resource into the well-being programs and policy response at RMH as one of a suite of support options for health care workers. Industry best practice standards for personal health information and data security will be followed. Data will be kept secure using industry-standard encryption over the wire and at rest. Regulations to host data in Australia will be followed, and data security measures will comply with the Open Web Application Security Project’s health care guidelines, the Australian Privacy Act, the ISO AS/NZ 27001, ISO AS/NZ 27017, and ISO AS/NZ 27018 standards, and SOC 2. The outcome paper will present a more detailed architecture in accordance with guidelines for reporting of health interventions using mobile phones [29].

### Implementation

The RMHive app will be implemented for use by health care workers hospital-wide. An implementation strategy will be developed to guide the wider hospital release, and the effectiveness of this strategy in supporting the engagement, adoption, integration, and future sustainability of the app into individual work and organizational culture will be reported on in the impact evaluation. A 12-week impact evaluation period has been set following the release of the MVP to assess its uptake, engagement, use patterns, and impacts on mental health. This includes an assessment of the implementation enablers and challenges and the integration of data from the design and development stages that will inform the three impact evaluation questions.

#### Participants and Eligibility

Health care workers in the emergency and cardiology departments will participate in the preparatory phase, the adaptive governance structures and frameworks, and the planned design and development processes. Up to 30 health care workers will provide photo interview data and participate in deliberative workshops (including subsequent developer-conducted co-design workshops), and up to 15 health care workers will participate in think aloud interviews to inform the beta test. Governance interviews will be completed by up to 30 managers and leaders through the hospital. For wider release and implementation, all health care workers at RMH will be able to access the app and download it for use. Users will be provided an access code. In terms of understanding use and engagement with the app, 20% of health care workers who use the app for at least 7 to 30 days will be invited to participate in a think aloud interview. For the identification of the mental health needs of health care workers and the impact of the app use on their mental health, only data collected from the MVP stage will be included.

#### Sample Size

Up to 10,000 health care workers at RMH will have access to the RMHive app. Our sample size of app users is calculated based on an anticipated minimum rate of 10% of total staff downloading RMHive (n=1000). Of these users, 50% may go on to use the app (n=500), and of these, a further 50% are anticipated to cease using the app within the first 5 days (n=250). Of the 250 users remaining beyond five days, an additional 30% are anticipated to continue using the app for 7 days or more (n=175), and a further 50% of these users are likely to use the app for the full 30-day use period (n=87). These figures are based on download and use patterns reported in other health-related mobile apps [30].

#### Harms

Because the RMHive app development process will involve discussions and content about mental health and work challenges, there may be some risks to participant well-being. A protocolized participant distress response has been developed to support the needs of the participants, and study communications will provide avenues to national and institutional support services. Although RMHive is being developed to support the mental health of health care workers, the MVP is not a clinical or triaging tool, and it will not be developed to treat clinically determined symptoms of mental illness for this stage. Participants will be informed of this and will be encouraged both within the app and in study communications to seek further professional help if they feel their mental health and well-being needs warrant it. Some of the messages and advice within the app will be tailored to mental health screening outcomes (PHQ2 and GAD2) and provide directions to seek assistance where warranted. Recommended actions for the K10 results for self-monitoring will be included to guide access to professional support services. Support information will be made available in study communications through the research and development process and within the app.

### Impact Evaluation Procedure

#### Conceptual Framework

The RMHive development process will use EBCD concepts to embed the lived experience of health care workers within the app content and features and will use adaptive governance frameworks to share power arrangements and decision-making in the co-design and implementation of the app. This means that health care workers will be embedded at all stages and processes of development. It is anticipated that the integration of health care worker perspectives using an EBCD and adaptive governance approach will increase the engagement and ongoing use of the RMHive app and subsequently lead to sustainability for organizational use. Additionally, developmental processes that seek to engage and understand organizational governance efforts will increase the ability to present RMHive as a key component of the health and well-being strategy across the organization. The success of this integration and support from governance stakeholders will be evaluated with attention to the adaptive governance framework underpinning the research collaboration [19].

#### Impact Evaluation Questions

The three impact evaluation questions are outlined again in Table 1 alongside the data sources to inform these questions and the reporting or analytical framework proposed to answer the question. Further explanations of the planned analyses for the data sources are then described.

**Table 1:** Impact evaluation questions, data sources, and planned analyses.

Impact evaluation question	Data sources	Analytical focus or framework
1. What is health care workers’ engagement with RMHive, including use patterns, perceptions of content, and overall level of engagement?	Change log from beta testing to the minimally viable product User demographics and mental health baseline measures (PHQ4^a^, PHQ2^b^, GAD2^c^, K10^d^ self-monitoring and general health self-tracking questionnaires) App analytics data (bounce rates and patterns of use, including total time using and content use) Qualitative think aloud semistructured interview text and video data from the beta test and implementation phase	Descriptive overview of beta test engagement and content changes Descriptive statistics on user demographics (age, gender, profession), mental health baseline scores and averages, K10 first completion averages Number of app downloads, use patterns, content accessed; patterns of content engaged with, number of videos watched, time spent on content (where possible) Thematic analysis of think aloud interview text content examining user perceptions of the app and content and video analysis for app usability and feature engagement (attention to facial gestures, body language, and user workflow); these data will be considered against the touchpoints identified in the photo interviews and deliberative workshops to evaluate the question of whether the app meets the needs of health care workers
2. What contextual, sociotechnical, organizational, and individual features support or hinder implementation of the RMHive app?	Qualitative governance interview data with leaders in the hospital setting Touchpoints that emerged from the photo interviews and deliberative workshops during design and development that were related to contextual, sociotechnical, organizational, and individual barriers and facilitators for implementation; review of available mental health and well-being programs at the hospital Qualitative think aloud semistructured interview text and video analysis Web-based implementation survey of team leaders, managers, and other appropriate staff distributed via hospital contacts	NPT^e^ using the four NPT constructs to code interview, mapping, and brief survey data according to coherence (understanding of the problem—how people make sense of the mental health needs and well-being of health care workers, and the role of a mobile app in providing support), cognitive participation (engagement—how is the new technology driven forward, who buys in to it, and how is practice sustained), collective action (integration of new technology, skill set fit, integration of new technology, work done to operationalize and contextually execute new technology), and reflexive monitoring (how do groups and individuals start to assess whether a new approach or practice is working and what reconfigurations are undertaken by them to embed change) Identification of themes at the different levels in the qualitative interview data and deliberative workshop related to what supports or hinders app implementation and integration; these will also be mapped to NPT where appropriate Summary findings from a brief survey of managers and team leaders regarding the implementation of the mobile app
3. What are the identifiable impacts on the mental health of individual health care workers through adoption, implementation, and use of the RMHive app?	User demographics and mental health post–app use measures of depression, anxiety, and overall PHQ4 mental health score Self-monitoring data using K10 for psychological distress General health self-tracking questions: physical activity, relationships, productivity, and well-being	Age, gender, and professional role where available; pre-PHQ2, GAD2, and overall PHQ4 scores compared with post–app use scores (defined as 30 days or last mental health entry on screening questionnaires) K10 self-monitoring scores at the first time of app use and last user completion First and last entries of self-tracking general health questions Case studies of patterns for K10 and the four general questions for further exploration of user mental health patterns over time if relevant

^a^PHQ4: 4-item Patient Health Questionnaire.

^b^PHQ2: 2-item Patient Health Questionnaire.

^c^GAD2: 2-item Generalized Anxiety Scale.

^d^K10: Kessler Psychological Distress Scale.

^e^NPT: normalization process theory.

### Data Analysis Plan

#### Use Patterns (Impact Evaluation Questions 1 and 3)

Participation rates (eg, total app downloads and bounce rates) and demographic summaries of age, gender, and profession will be provided using descriptive statistics. User analytics will be described in terms of time using the app; engagement with video content (yes or no responses to whether content was helpful); frequency of accessing individual elements of the app; time spent watching video content; links to mental health support services; and number of uses within the evaluation period. The presentation of the technological aspect of RMHive development and implementation will follow the mobile health evidence reporting and assessment (mERA) reporting guidelines [29]. The user analytics overview is presented in the supplementary table in Multimedia Appendix 1.

#### Engagement With the App, Perceptions of Content, and Meeting Mental Health Needs (Impact Evaluation Question 1)

The photo interview and deliberative workshop text will be thematically analyzed using the Braun and Clarke approach, a theoretically flexible analysis method for qualitative data that draws out common patterns from data that relate to research questions [31]. This will enable themes related to mental health need identified in the needs analysis to be noted and considered against themes that may emerge from the discussion of use and content in the think aloud interviews through the beta testing and implementation of the MVP. Video analysis will examine body language, facial expressions, and discomfort or comfort with the use of the app to explore engagement with the app. Voice tone will be considered for user engagement.

#### Contextual, Sociotechnical, Organizational, and Individual Factors Affecting Implementation (Impact Evaluation Question 2)

The dynamic influence of contextual, sociotechnical, organizational, and individual factors and their impact on the implementation and engagement with RMHive will be assessed using normalization process theory (NPT) [32] and the mERA reporting guidelines [29]. NPT is an implementation science theoretical framework that is used to evaluate the success of implementation through a focus on actions rather than on beliefs or intentions [32]. NPT comprises four key constructs: coherence, which relates to the level of understanding people have about an intervention and the ways they make sense of new practices or technologies; cognitive participation, which describes the level of engagement and commitment people have to an intervention and the ways in which they start to embed or sustain a new practice or technology; collective action, which explores how well an intervention integrates with an organization’s goals and activities, sociotechnical workflows, and compatibility with existing practices; and reflexive monitoring, which relates to engagement in the appraisal and monitoring of the intervention and outcomes, including the extent to which individuals and groups reconfigure their practice to sustain new practices or technologies [33]. The NPT framework will be applied specifically to the governance interview content, the brief implementation survey, and the assessment of the implementation strategies developed for the MVP release. If relevant contextual or organizational themes are identified from the photo interviews and the beta test phase think aloud interviews, these will also be coded to the NPT constructs to support this analysis. Implementation leads (managers, team leaders, and health care worker members of the project team) will be provided with a link to a web-based survey to identify their awareness of RMHive, their use of the app, and any notable barriers or challenges that they have experienced. The facilitators and barriers of RMHive uptake will be examined at an individual level through engagement with app users and at an institutional level through ongoing governance interviews with respondents in management roles within RMH and the broader sociocultural context. mERA will be used to describe further technical implementation [29].

#### Impact on Mental Health (Impact Evaluation Question 3)

The impact evaluation will examine the profile of health care workers using RMHive, how RMHive is being used by health care workers through user analytics, and how the self-reported mental health of health care workers changes over the evaluation period.

On using the app for the first time, RMHive users will be prompted to establish a user profile and enter baseline data, including the PHQ4, which includes the PHQ2 and GAD2 subscales. The RMHive app will capture broad demographics, including age range; gender; whether the person is in a leadership position; and broad professional group (allied health; medical; nursing; administrative; environmental services; other). Additionally, users will be prompted to enter subjective general health ratings of their mood, physical health, productivity, and relationships on a 3-point scale (on track; neutral; not on track). Descriptive statistics will be used to summarize the sociodemographic and professional characteristics of the participants, their mental health responses, and subjective ratings collected at baseline and the last completed measure. For continuous data with a skewed distribution, medians and quartiles will be used instead.

The PHQ4 [26] will be the primary study outcome as an indicator of symptom burden, functional impairment, and disability, and RMHive users will be prompted to complete the PHQ4 at baseline, day 14, and day 28. The PHQ4 consists of the GAD2 anxiety subscale and the PHQ2 depression subscale. The PHQ2 assesses the presence of symptoms of depression over the last two weeks using a 4-point Likert scale (0, not at all; 1, several days; 2, more than half the days; 3, nearly every day). Total scores are calculated by summing the two items and can range between 0 and 6. The GAD2 assesses the presence of generalized anxiety symptoms over the past two weeks using a 4-point Likert scale (0, not at all; 1, several days; 2, more than half the days; 3, nearly every day). GAD2 has also been determined to indicate posttraumatic stress. Scores above 3 on each subscale will indicate symptoms of depression or anxiety [26,34,35]. The results of the two subscales will be reported individually and then summed to generate a PHQ4 score that can range from 0-12, with higher scores indicating an increased likelihood of underlying depressive or anxiety disorder.

Users will be provided with access to the 10-standard-item Kessler Psychological Distress Scale (K10) for self-monitoring upon completing their profile in the RMHive app [24]. Respondents will be asked to indicate how often in the past four weeks they have experienced certain symptoms (eg, nervousness, hopelessness, fatigue, agitation, and depressed mood) using a 5-point Likert scale (1, not at all, to 5, all the time). The total K10 score is the sum of the 10 items, ranging from 10-50, where higher K10 scores indicate greater higher psychological distress. If one item on the K10 is missing a response, the missing values will be substituted with the mean response of the completed items; otherwise, the total score will be coded as missing. Users will be able to self-monitor their emotional state at any time and will receive reminder prompts on day 2, day 5, and then weekly through app use. Completion of the K10 will be optional throughout the study, and users will be asked about the benefits or drawbacks of having access to the K10 for self-monitoring in the think aloud interviews for the wider release. K10 results will be reported using first and last completion of the measure by users. Subanalyses will be explored based on developing user case studies to examine over-time outcomes and self-monitoring trajectories.

Primary analysis will involve repeated measures analysis of covariance of the PHQ4 and subscale scores from baseline to day 14 and day 28. For users who only have baseline data, this analysis will be used to inform the question of the overall mental health need of health care workers. For users who enter data at baseline and again at both day 14 and day 28 or on either day, these time points will be reported as baseline, middle, and post use. For users with only two completed PHQ4 scores, these scores will be reported as pre and post. The analysis will progress with the existing data at each time point. For the secondary analysis, linear regression will be used to estimate the difference in the mean change from baseline in the mean K10 emotional state tracking and last use of K10.

### Data Handling

Deidentified data with unique record identifiers for each participant will be extracted from the data collection system in the form of comma-separated value (CSV) data files. All research data will be stored in a deidentified format and will only be accessible to named research team members involved in the analysis process approved by the ethics committee. Data transfers from the app to the evaluation team will be conducted weekly during the beta test and following the MVP release as CSV files. The project manager will then download the CSV data files and will save the dummy-coded files to the central password–protected university system, where they will be stored securely and backed up regularly. Aggregate user analytics will be extracted using Firebase, and queries will be analyzed through Google Analytics and exported to CSV files for reporting and analysis. The data manager will then import the CSV files into Stata 15 (StataCorp LLC) [36] for data processing and statistical analysis. Data will be checked to identify errors and, where possible, resolve them before the analyses are conducted. Steps will include labelling the variables and values, creating composite variables, and creating the total scores according to the instrument’s guidelines. Data sets will be merged using the unique identifier generated for each participant. Deidentified data will be stored on password-protected university servers with access limited to the research team for future use in accordance with the National Statement on Ethical Conduct in Human Research [37].

## Results

The RMHive program of work received funding in June 2020 and institutional ethics approval on June 9, 2020. Governance structures and committees were implemented in June, and data collection commenced in July. The impact evaluation will continue from design, development, and implementation up to mid-February 2021. It is anticipated that the study outcomes will be published in mid-2021.

## Discussion

The adverse impact of pandemics on the mental health of health care workers has been well established [2,3], and prior research has established that mental health supports that address the needs of health care workers are required [13]. To date, the majority of mental health support tools for health care workers addressing pandemics have not incorporated the lived experience of end users in their development or implementation [13]. This may be a reason for the limited uptake and engagement with mobile apps. Mental health support tools developed and deployed in a compressed time frame and without an understanding of the lived experience of health care workers and their mental health needs carry an increased risk of not being delivered in a format that is readily accessible, desired, or ultimately used by health care workers. RMHive seeks to address these risks by using EBCD to support clinicians and researchers working together in a process of shared decision-making and co-design, leading to an app that centers the lived experience of health care workers as a basis for responding to their mental health needs [10]. This further extends the HCD approach to ensure active co-design by people with lived experience and shared power.

The RMHive app will be further supported by an impact evaluation that will provide critical insights into the contextual, sociotechnical, organizational, and individual factors that contribute to its implementation, engagement, and use. The impact evaluation [38] will expand current digital health frameworks by providing new insights into how EBCD processes inform the design, development, and implementation of an app directed toward addressing the mental health needs of health care workers. In keeping with the impact evaluation method, a theory of change will be produced from the evaluation to inform the future rollout and wider use of the app as a possible mental health and well-being intervention or support program. The impact of RMHive on the mental health outcomes of health care workers will also be assessed. It is recognized that in this evaluation, we are only focusing on near impacts of the RMHive app within the implementation context, and it is possible that the evaluation timeframe may not be sufficient for longer-term individual- and organizational-level changes to be observed.

To our knowledge, RMHive is the first mobile app developed using EBCD to support the mental health of health care workers in response to a pandemic. It is hoped that RMHive will be a valuable support through the COVID-19 pandemic for health care workers who are experiencing increased challenges to their mental health and well-being. The impact evaluation outcomes will provide a valuable addition to local and international efforts to support the mental health of health care workers through the deployment of digital mental health tools that can be rapidly co-designed and scaled in response to major events such as a global pandemic.

## Appendix

Multimedia Appendix 1 Supplementary table: overview of the user analytics plan.

## Abbreviations

COVIDDA: COVID Digital Asset
CSV: comma-separated value
EBCD: experience-based co-design
GAD2: 2-item Generalized Anxiety Scale
HCD: human-centered design
K10: Kessler Psychological Distress Scale
mERA: mobile health evidence reporting and assessment
MVP: minimally viable product
NPT: normalization process theory
PHQ4: 4-item Patient Health Questionnaire
PHQ2: 2-item Patient Health Questionnaire
RMH: Royal Melbourne Hospital
