# Supplementary material for: Rapid Design and Delivery of an Experience-Based Co-designed Mobile App to Support the Mental Health Needs of Health Care Workers Affected by the COVID-19 Pandemic: Impact Evaluation Protocol
Source: JMIR Res Protoc. 2021 Mar 9;10(3):e26168. doi: 10.2196/26168 (PMC7945974; doi:10.2196/26168)
Supplement: Multimedia Appendix 1 [file resprot_v10i3e26168_app1.docx]

**Supplementary Table 1. Overview of user analytics plan.**

| **User analytics** | **Definition** |
| --- | --- |
| Total number of users | Total number of RMHIVE users |
| New versus returning users | Proportion of first time versus continuing RMHIVE users |
| Bounce rate (all users) | Average number of bounces across all pages divided by the total number of visits across all those pages within the study period |
| Bounce rate (per page) | Total number of bounces divided by the total number of visits on a page. |
| Average session duration (all users) | Average session duration for all users |
| Average session duration (per user) | Average session duration per user |
| Average events per session | Average number of tasks (video views, survey completion) per session |
| Average number of pageviews per page | Average number of pageviews for each webpage |
| Average number of pageviews per user | Average number of pageviews for each user |
| Proportion of users completing the survey | Number of survey completers divided by the total number of RMHIVE app users (survey completers + non-completers) |
| Proportion of users playing at least one video in a session | Number of video viewers divided by the total number of RMHIVE app users (viewers + non-viewers) |
| Average number of videos viewed (all users) | Average number of videos viewed for all RMHIVE users |
| Average number of views per video | Average number of times each video is viewed for all RMHIVE users |
